# Comparative analysis of ChatGPT and Gemini (Bard) in medical inquiry: a scoping review

**DOI:** 10.3389/fdgth.2025.1482712

**Published:** 2025-02-03

**Authors:** Fattah H. Fattah, Abdulwahid M. Salih, Ameer M. Salih, Saywan K. Asaad, Abdullah K. Ghafour, Rawa Bapir, Berun A. Abdalla, Snur Othman, Sasan M. Ahmed, Sabah Jalal Hasan, Yousif M. Mahmood, Fahmi H. Kakamad

**Affiliations:** ^1^ Scientific Affairs Department, Smart Health Tower, Sulaymaniyah, Iraq; ^2^ College of Medicine, University of Sulaimani, Sulaymaniyah, Iraq; ^3^ Civil Engineering Department, College of Engineering, University of Sulaimani, Sulaymaniyah, Iraq; ^4^ Department of Urology, Sulaimani Surgical Teaching Hospital, Sulaymaniyah, Iraq; ^5^ Kscien Organization for Scientific Research (Middle East Office), Sulaymaniyah, Iraq

**Keywords:** ChatGPT, Google Bard, medical inquiries, comparison, madical AI

## Abstract

**Introduction:**

Artificial intelligence and machine learning are popular interconnected technologies. AI chatbots like ChatGPT and Gemini show considerable promise in medical inquiries. This scoping review aims to assess the accuracy and response length (in characters) of ChatGPT and Gemini in medical applications.

**Methods:**

The eligible databases were searched to find studies published in English from January 1 to October 20, 2023. The inclusion criteria consisted of studies that focused on using AI in medicine and assessed outcomes based on the accuracy and character count (length) of ChatGPT and Gemini. Data collected from the studies included the first author's name, the country where the study was conducted, the type of study design, publication year, sample size, medical speciality, and the accuracy and response length.

**Results:**

The initial search identified 64 papers, with 11 meeting the inclusion criteria, involving 1,177 samples. ChatGPT showed higher accuracy in radiology (87.43% vs. Gemini's 71%) and shorter responses (907 vs. 1,428 characters). Similar trends were noted in other specialties. However, Gemini outperformed ChatGPT in emergency scenarios (87% vs. 77%) and in renal diets with low potassium and high phosphorus (79% vs. 60% and 100% vs. 77%). Statistical analysis confirms that ChatGPT has greater accuracy and shorter responses than Gemini in medical studies, with a *p*-value of <.001 for both metrics.

**Conclusion:**

This Scoping review suggests that ChatGPT may demonstrate higher accuracy and provide shorter responses than Gemini in medical studies.

## Introduction

Artificial Intelligence (AI) and Machine Learning (ML) are interconnected technologies recently gaining significant popularity. AI involves creating intelligent machines capable of performing tasks that typically require human intelligence, such as visual perception, speech recognition, decision-making, and language translation. ML, a subset of AI, focuses on developing algorithms and statistical models that enable machines to learn from data and improve their performance over time without explicit programming (1, 2). AI is at the forefront of transforming various aspects of our lives by altering how we analyze information and enhancing decision-making through problem-solving, reasoning, and learning (3).

In the dynamic domain of AI chatbots, the comparative analysis of ChatGPT and Gemini (formerly known as Google's Bard) has emerged as a focal point, particularly in medical inquiries (1, 2). Recent investigations have explored the precision and effectiveness of these AI models in fielding medical questions across various specialties (1, 4–7). These studies demonstrate ChatGPT's capabilities in diagnostic imaging and clinical decision support, underscoring its potential value in healthcare settings (4–6).

In recent years, AI models like ChatGPT and Gemini have significantly impacted natural language processing, particularly in healthcare. ChatGPT, developed by OpenAI, provides relevant and accurate text-based responses using a large dataset (1, 5). While Gemini, from Google DeepMind, integrates multimodal capabilities, handling text, audio, and video, which is especially useful in medical imaging (5). However, both models face challenges. ChatGPT, for example, shows variability in psychiatric assessments and struggles with complex cases (2). Additionally, AI models still struggle to interpret nuanced human emotions and contexts (4).

While AI chatbots like ChatGPT and Gemini show promise in medicine, extensive research is still required to understand their capabilities properly. It is essential to address the variation in their performance across different medical scenarios and enhance their accuracy for various medical applications (8). The use of AI in healthcare faces several challenges, including data privacy, algorithm accuracy, adherence to ethical standards, societal acceptance, and clinical integration (9, 10). These challenges make it difficult to develop precise and reliable AI systems. Privacy concerns restrict access to relevant data, and potential biases can result in inaccurate outcomes (8, 10).

This scoping review aims to evaluate and compare the accuracy and length of ChatGPT and Gemini (Google's Bard) in addressing medical inquiries across diverse fields, focusing on their strengths, limitations, and practical implications for healthcare. As AI models become increasingly integrated into clinical and educational settings, understanding their performance variability is essential. Both models face challenges, including inconsistencies in complex cases, privacy concerns, and ethical issues. This review offers insights to help researchers, practitioners, and developers optimize these tools for more effective decision-making and patient care.

## Methods

### Study protocols

We applied a systematic approach to assess the methodological quality of our scoping review, including comprehensive literature searches, double screening, bias assessment, and evaluation of publication bias.

### Data sources and search strategy

A systematic search was conducted in databases and search engines, including Google Scholar, PubMed/MEDLINE, Cochrane Library, Web of Science, CINAHL, and EMBASE, using keywords such as (“ChatGPT” OR “GPT-3” OR “GPT-4” OR “Bard” OR “Gemini”) AND (“Medical” OR “Healthcare” OR “Clinical” OR “Health Inquiry” OR “Medical Inquiry”) AND (“comparison” OR “comparative” OR “analysis” OR “review”) to identify studies published from January 1 to October 20, 2023. The search was restricted to studies published in English and related to human health subjects.

### Eligibility criteria

To be included in this study, studies needed to meet the following criteria: focus on the application of ChatGPT and Gemini across different branch specialties, evaluate outcomes based on the accuracy and character count of ChatGPT and Gemini, and be verified against the most recent predatory journal list (11). Additionally, review articles and case reports were excluded.

### Study selection process

The initial screening involved two researchers reviewing all titles and abstracts to check if they met the eligibility criteria. In case of disagreements, a third author was consulted to reach a final decision and resolve conflicts between the initial researchers.

### Data items

The data collected from the studies included the first author's name, country of study, type of study design, publishing year, sample size, type of medical specialty, accuracy, and length (character) of ChatGPT and Gemini. Accuracy refers to the ability of ChatGPT and Gemini to provide contextually appropriate and correct responses to medical questions based on the standard guidelines specific to each medical specialty.

### Data analysis and synthesis

Microsoft Excel (2019) was utilized to collect and organize the extracted data, while descriptive analysis was conducted using the Statistical Package for Social Sciences (SPSS) software (version 26). The data is displayed as frequencies, percentages, means, and standard deviations.

## Results

### Study selection

**Figure 1:**
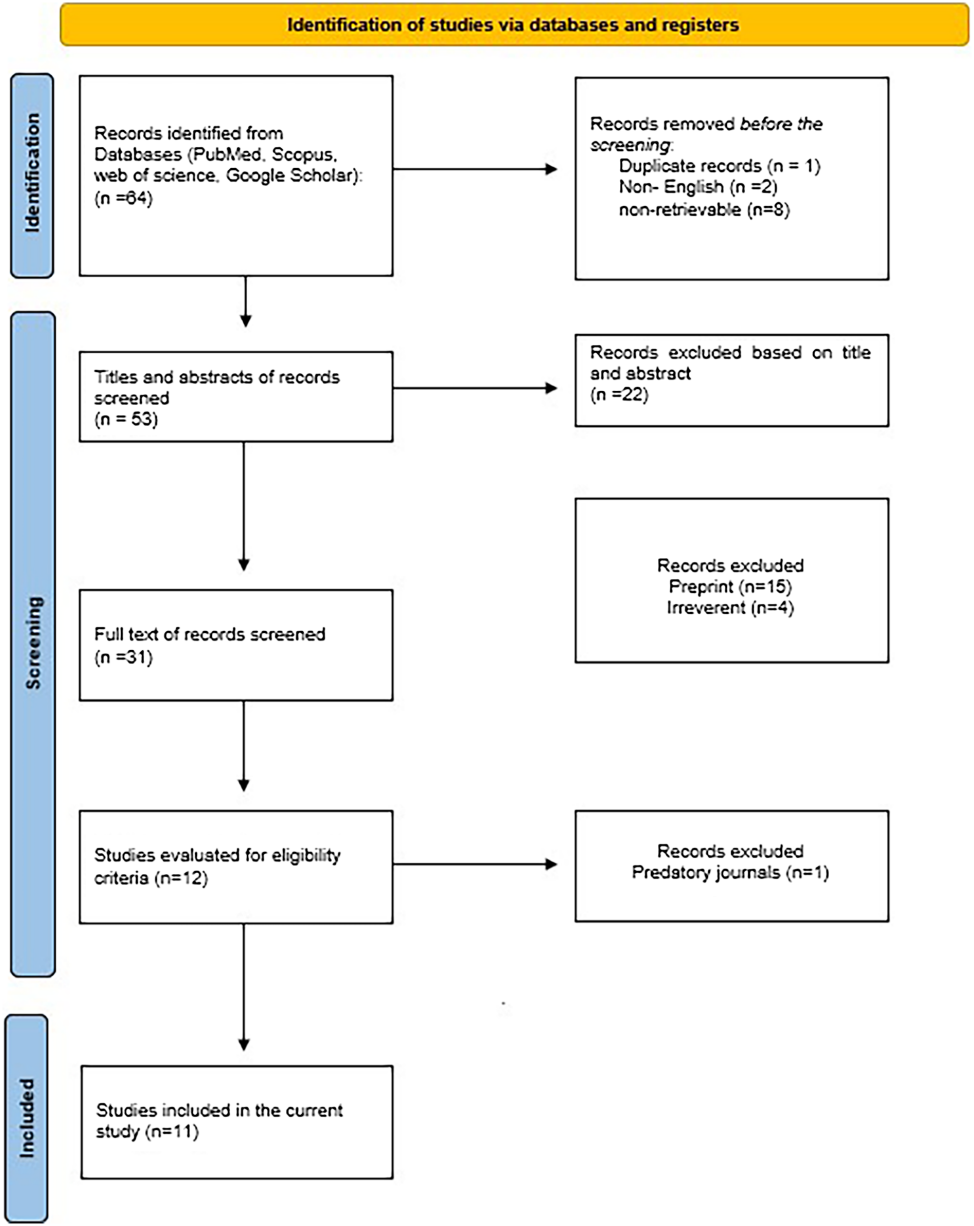
Prisma flow diagram.

During the initial database search, a total of 64 articles were identified. Pre-screening procedures removed one duplicate, two articles in non-English languages, and eight with unretrievable data. Following a comprehensive review of titles and abstracts, 53 studies were assessed, excluding 22 for lack of relevance. The remaining 31 studies underwent full-text evaluation, excluding 19 for failing to meet the inclusion criteria. Among the 12 studies that proceeded to the eligibility assessment phase, one was excluded due to its publication in a predatory journal. Ultimately, 11 studies met the criteria for inclusion in the review (Figure 1).

### Characteristics of the included studies

The summarized raw data from the included studies are all observational in Tables 1, 2. India and the United States were the primary contributors, providing two studies. Additionally, Canada, Singapore, Turkey, Australia, Ecuador, and Iraq each contributed one study (Table 1).

**Table 1:** Baseline characteristics of the included studies.

No	Author	Type of study	Publishing years	Country	Sample size	Specialty
1	Patil (12)	Cross-sectional	2023	Canada	29 (9.12%)	Neuroradiology
					19 (5.97%)	Mammography
					89 (27.99%)	General & physics
					30 (9.43%)	Nuclear medicine
					16 (5.03%)	Pediatric Radiology
					26 (8.18%)	Interventional radiology
					29 (9.12%)	Gastrointestinal radiology
					11 (3.46%)	Genitourinary radiology
					16 (5.03%)	Cardiac radiology
					6 (1.89%)	Chest radiology
					25 (7.86%)	Musculoskeletal radiology
					22 (6.92%)	Ultrasound
2	Kumari (13)	Cross-sectional	2023	India	50	Hematology
3	Dhanvijay (14)	Cross-sectional	2023	India	77	Physiology
4	Muhialdeen (15)	Cross-sectional	2023	Iraq	20	Clinical Diagnosis
5	Koga (16)	Cross-sectional	2023	USA	25	neurodegenerative disorders
6	Zhi Wei Lim (17)	Cross-sectional	2023	Singapore	31	myopia care
7	Ilgaz (18)	Cross-sectional	2023	Turkey	131	Anatomy
8	Seth (19)	Cross-sectional	2023	Australia	6	Rhinoplasty
9	Salazar (20)	Cross-sectional	2023	Ecuador	75	Emergency
					101	Non-emergency
10	Qarajeh (21)	Cross-sectional	2023	USA	81 (33.75%)	Renal Diet High potassium
					68 (28.33%)	Renal Diet Low potassium
					91 (37.91%)	Renal Diet High phosphorus
11	Toyama (22)	Cross-sectional	2023	Japan	103	Radiology

**Table 2:** Comparison between ChatGPT and Bard.

No	Author	Specialty	ChatGPT accurate	Gemini accurate	ChatGPT length (character)	Gemini length (character
1	Patil (12)^a^	Neuroradiology	100.00%	86.21%	840.90 (±426.35)	1,443.52 (±415.88)
		Mammography	84.21%	68.42%	787.63 (±447.38)	1,454.95 (±442.34)
		General & physics	85.39%	68.54%	1,022.38 (±453.50)	1,490.69 (±406.58)
		Nuclear medicine	80.00%	56.67%	947.30 (±486.57)	1,321.57 (±374.86)
		Pediatric Radiology	93.75%	68.75%	764.63 (±330.04)	1,368.88 (±547.91)
		Interventional radiology	88.46%	80.77%	952.31 (±510.00)	1,538.31 (±446.90)
		Gastrointestinal radiology	89.66%	79.31%	901.93 (±423.01)	1,427.66 (±322.21)
		Genitourinary radiology	72.73%	63.64%	1,048.82 (±338.28)	1,373.09 (±342.2)
		Cardiac radiology	75.00%	68.75%	915.50 (±3.48)	1,537.94 (±692.44)
		Chest radiology	100.00%	83.33%	816.33 (±303.77)	1,492.33 (±295.28)
		Musculoskeletal radiology	80.00%	64.00%	945.48 (±394.93)	1,326.40 (±316.33)
		Ultrasound	100.00%	63.64%	944.91 (±518.11)	1,371.95 (±352.66)
2	Kumari (13)^b^	Hematology	3.15/5 (63%)^A^	2.23/5 (44%)^A^	-	-
3	Dhanvijy (14)^b^	Physiology	3.19/4 (79%)^A^	2.15/4 (53%)^A^	-	-
4	Muhialdeen (15)^b^	Clinical Diagnosis	90%	80%	-	-
5	Koga (16)^a^	neurodegenerative disorders	84%	76%	-	-
6	Zhi Wei Lim (17)^a^	myopia care	80.6%	54.8%	1,221.13 (±323.32)	1,275.87 (±393.25)
7	Ilgaz (18)^b^	Anatomy	44.27%	41.98%	-	-
8	Seth (19)^b^	Rhinoplasty	4/5 (80%)^A^	4/5 (80%)^A^	-	-
9	Salazar (20)^b^	Emergency	77%	87%	-	-
		Non-emergency	36%	33%	-	-
10	Qarajeh (21)^a^	Renal Diet High potassium	99%	79%	-	-
		Renal Diet Low potassium	60%	79%	-	-
		Renal Diet high phosphorus	77%	100%	-	-
11	Toyama (22)^a^	Radiology	65%	39%	-	-

^a^ ChatGPT-4.

^b^ ChatGPT-3.5.

### Main findings

The research included 1,177 samples from 11 different medical specialties (12–22). In radiology, there were 421 samples, encompassing various fields such as neuroradiology (9.12%), mammography (5.97%), general and physics (27.99%), nuclear medicine (9.43%), pediatric radiology (5.03%), interventional radiology (8.18%), gastrointestinal radiology (9.12%), genitourinary radiology (3.46%), cardiac radiology (5.03%), chest radiology (1.89%), musculoskeletal radiology (7.86%), and ultrasound (6.92%). The renal sample size was 240, divided into the renal diet with high potassium (33.75%), the renal diet with low potassium (28.33%), and the renal diet with high phosphorus (37.91%). Emergency and non-emergency cases had sample sizes of 176, while the smallest samples were in clinical diagnosis and neurodegenerative disorders, with sizes of 20 and 25, respectively (Table 1).

The comparison between ChatGPT and Gemini across various specialties reveals accuracy and response length differences. ChatGPT generally may demonstrate higher accuracy than Gemini, especially in radiology specialties. The average accuracy of ChatGPT was 87.43%, higher than Gemini 71%. Additionally, the average response length of ChatGPT was 907 characters, shorter than Gemini's 1,428 characters. This indicates that ChatGPT's accuracy relative to response length may be more reliable and accurate than Gemini. Accuracy in the hematology specialty, ChatGPT, was 63%, compared to Gemini's 44%. Similar trends are observed in physiology, clinical diagnosis, neurodegenerative disorders, anatomy, renal diet, high potassium, and radiology. Conversely, in myopia care, the response lengths of ChatGPT and Gemini were nearly the same (1,221.13 vs. 1,275.87), with ChatGPT achieving a higher accuracy of 80.6% compared to Gemini's 54.8%. In rhinoplasty, both ChatGPT and Gemini demonstrate the same accuracy. In contrast, Gemini may be more accurate than ChatGPT in emergency scenarios, a renal diet with low potassium and a renal diet with high phosphorus (87% vs. 77%, 79% vs. 60%, and 100% vs. 77%, respectively) (Table 2).

The statistical analysis compared the accuracy and response length of ChatGPT and Gemini. The results indicate that ChatGPT has a higher accuracy (72.06%) than Gemini (63.38%), with a mean difference of 8.68, a confidence interval of 7.77–9.58, and a statistically significant *p*-value of <.001. In terms of response length, ChatGPT produces shorter responses (960.84 words) compared to Gemini (1,423.15 words), with a mean difference of 462.31 and a similarly significant *p*-value of <.001. This statistical comparison emphasizes that ChatGPT may be more accurate and generates shorter responses than Gemini (Table 3).

**Table 3:** Statistical analysis of ChatGPT and Gemini.

	Mean	Mean difference	Std. deviation	Confidence interval		*p*-value
				Lower	Upper	
ChatGPT Accuracy	72.06	8.68	15.82	7.77	9.58	<.001
Gemini Accuracy	63.38					
ChatGPT Length	960.84	462.31	158.10	445.64	478.98	<.001
Gemini Length	1,423.15					

## Discussion

Implementing large language models (LLMs) in medical education shows significant potential for transforming traditional teaching methods. Models like ChatGPT and Gemini process extensive medical literature, providing valuable, contextually relevant information for educators and students (23, 24). LLMs create interactive, dynamic learning by giving students access to current medical data, clarifying complex concepts, and enhancing problem-solving. They also improve knowledge retrieval and support evidence-based decision-making. Incorporating LLMs encourages self-directed learning, critical thinking, and ongoing professional growth. However, recognizing their limitations and biases is essential for responsible, ethical use, complemented by practical training and clinical mentorship (25, 26).

AI models have demonstrated significant potential in assisting medical professionals by enhancing efficiency in problem-solving, diagnosis, and data interpretation. For instance, ChatGPT has consistently outperformed models like Bard and Bing in accuracy when addressing medical vignettes (13, 14). This underscores AI's pivotal role in supporting clinical decision-making, particularly in complex fields such as hematology (13). However, despite these encouraging results, AI models still face limitations, including inconsistencies in performance across various medical specialties, which necessitate further refinement before full integration into clinical practice (21).

The comparative analysis of ChatGPT and Gemini across various medical specialties reveals distinct patterns in their accuracy and response length performance. ChatGPT consistently demonstrates higher accuracy rates compared to Gemini in most specialties. This trend is evident in specialties such as neuroradiology (100% vs. 86.21%), hematology (63% vs. 44%), physiology (79% vs. 53%), clinical diagnosis (90% vs. 80%) and neurodegenerative disorders (84% vs. 76%). ChatGPT's superior accuracy indicates its potential as a reliable tool for medical inquiries, providing precise and dependable information across various medical fields (12–16).

Despite Gemini's lower accuracy rates, it consistently delivers longer responses than ChatGPT. In neuroradiology, Gemini's responses averaged 1,443.52 characters compared to ChatGPT's 840.90 characters. This pattern is repeated across other specialties, such as mammography (1,454.95 vs. 787.63) and general & physics (1,490.69 vs. 1,022.38) (12).

The longer response length of Gemini suggests that it may offer more detailed and comprehensive information, which could be beneficial in scenarios where a more exhaustive explanation is needed. While comparing ChatGPT and Gemini for accuracy and response length in chest radiology and ultrasound, ChatGPT consistently outperforms Gemini in accuracy. ChatGPT achieves 100% accuracy for chest radiology compared to Gemini's 83.33%, with a shorter average response length of 816.33 vs. Gemini's 1,492.33 characters. ChatGPT also has a perfect accuracy rate of 100% in ultrasound, while Gemini's accuracy drops to 63.64%. Similarly, ChatGPT's responses are more concise, averaging 944.91 characters compared to Gemini's 1,371.95 characters (12).

ChatGPT and Gemini in myopia care respond to similar lengths (1,221.13 and 1,275.87 characters, respectively). However, there is a difference in accuracy: ChatGPT achieves an accuracy of 80.6%, whereas Gemini achieves 54.8%. This disparity suggests that while both models may offer comparable responses in terms of content, ChatGPT tends to provide more reliable and accurate information in this specialized medical context (17).

ChatGPT and Gemini exhibit nearly identical accuracy in anatomy and rhinoplasty. ChatGPT achieves 44.27% accuracy in anatomy, slightly higher than Gemini's 41.98%. For rhinoplasty, both models perform equally well, each with an accuracy rate of 80%. This comparison demonstrates that ChatGPT and Gemini perform similarly in these medical specialties (18, 19).

Exceptions to this trend were observed in emergency scenarios, where Gemini achieved higher accuracy (87%) compared to ChatGPT (77%) (20). This highlights that Gemini may have strengths in specific contexts, such as emergencies where detailed information could be critical. However, both models showed lower accuracy rates in non-emergency scenarios, with ChatGPT slightly outperforming Gemini (36% vs. 33%) (20).

The performance of ChatGPT and Gemini in providing dietary advice for renal conditions also varied. ChatGPT excelled in high potassium contexts (99% vs. 79%) but was less accurate in low potassium and high phosphorus scenarios compared to Gemini (77% vs. 100%) (21). This variability suggests that each model may have specialized strengths in specific medical contexts, and their combined use could potentially enhance the quality of medical inquiry responses.

The comparative analysis of ChatGPT and Gemini (Bard) in medical inquiry highlights several limitations. ChatGPT may provide inaccurate medical information due to its limited understanding of complex contexts, and biases in training data can affect accuracy. Ethical concerns include the risk of outdated information and issues related to patient data privacy. Additionally, the evolving nature of large language models means that ChatGPT and Gemini are frequently updated, potentially rendering some findings obsolete as newer versions are released. The study's focus on specific models and predefined case vignettes may restrict its findings, as the scope of medical inquiries is limited to particular scenarios, which may not fully capture the broad range of medical topics these models could encounter. Moreover, potential biases in the responses of these language models were not fully explored, affecting the generalizability of the results. There may be limitations and potential bias in measuring accuracy, as each specialty uses different standard answers to compare with the responses of ChatGPT and Gemini across various studies. This variability makes it challenging to determine how accurately the models perform in each specialty. The findings indicate that ChatGPT generally offers more accurate and concise responses across various medical specialties, while Gemini provides more detailed but less accurate answers. The choice between these AI models should be guided by the specific needs of the medical inquiry—whether precision or detail is prioritized. Future improvements should aim to integrate the strengths of both models, enhancing accuracy while maintaining the comprehensiveness of responses to support better clinical decision-making and patient care.

## Conclusion

This scoping review indicates that ChatGPT has shown promise in the included medical studies. It may demonstrate higher accuracy and a shorter response than Gemini. Therefore, further research is needed to maximize ChatGPT's accuracy compared to Gemini in the medical field.

## Data Availability

The original contributions presented in the study are included in the article/Supplementary Material, further inquiries can be directed to the corresponding author.
